# Cognitive judgment bias in the psychostimulant-induced model of mania in rats

**DOI:** 10.1007/s00213-014-3707-y

**Published:** 2014-08-14

**Authors:** Rafal Rygula, Ewa Szczech, Jakub Kregiel, Joanna Golebiowska, Jakub Kubik, Piotr Popik

**Affiliations:** 1 Affective Cognitive Neuroscience Laboratory, Department of Behavioral Neuroscience and Drug Development, Institute of Pharmacology Polish Academy of Sciences, 12 Smetna Street, 31-343 Krakow, Poland; 2 Faculty of Health Sciences, Collegium Medicum, Jagiellonian University, Michalowskiego 12, 31-126 Krakow, Poland

**Keywords:** Rat, Cognitive judgment bias, Ambiguous cue, Amphetamine, Cocaine, Mania, Bipolar disorder, Behavioral, Animal model

## Abstract

**Rationale:**

Animal models of mania lack genuine cognitive parameters. The present gold standard of mania models, amphetamine-induced hyperlocomotion, is rather unspecific and does not necessarily target its cardinal symptoms. Therefore, alternative behavioral markers that are sensitive to stimulants are required.

**Objectives:**

In the present study, by combining the psychostimulant-induced model of mania in rodents with the recently developed ambiguous-cue interpretation (ACI) tests, we investigated the effects of chronic administration of d-amphetamine and cocaine on the cognitive judgment bias of rats.

**Methods:**

To accomplish this goal, in two separate experiments, previously trained animals received chronic, daily injections of either d-amphetamine (2 mg/kg) or cocaine (10 mg/kg) for 2 weeks and were subsequently tested with the ACI procedure.

**Results:**

Chronic treatment with both psychostimulants did not make rats more “optimistic.”

**Conclusions:**

The results are discussed in terms of behavioral and pharmacological actions of the tested compounds and their implications for modeling mania in animals.

## Introduction

Mania is a debilitating psychiatric condition and a cardinal feature of bipolar disorder (BD). Critical features of mania include over-excitement, grand or extravagant style, expanded self-esteem, pressured speech, reduced need for sleep, over-indulgence in enjoyable activities, and over-optimistic judgment bias leading to high-risk behaviors, such as extravagant shopping, sexual adventures, or improbable commercial schemes (Belmaker and Bersudsky 2004; Young et al. 2011a). Clinical studies have highlighted the importance of excessive optimism in this disorder (Carver and Johnson 2009; Gamma et al. 2008; Giovanelli et al. 2013; Johnson 2005; Johnson and Jones 2009; Leahy 1999), and Beck and Weishaar (1995) argued that “overly optimistic expectations provide vast sources of energy and drive the manic individual into continuous goal-directed activity.”

As BD affects 2 to 7 % (including subsyndromal manic symptom group) of the population (Angst et al. 2003; Judd and Akiskal 2003; Kessler et al. 2005; Merikangas et al. 2007; Schaffer et al. 2006), this disease needs to be better understood, and new treatment strategies need to be developed. These goals can be achieved by establishing new, preclinical animal models with translational validity.

Modeling BD mania in animals began with observational evidence that psychostimulants (such as amphetamine) can produce mania-like symptoms in normal healthy subjects (Peet and Peters 1995) and exacerbate symptoms or induce a manic episode in patients (Meyendorff et al. 1985). The effects of psychostimulants on behavior have therefore been widely used as an animal model of mania (Frey et al. 2006a, e; Kato et al. 2007). Because psychomotor agitation is commonly observed during mania and locomotor activity is easily measured in rodents, amphetamine-induced hyperactivity became the “gold-standard” rodent model of mania. The use of this model does have however, multiple limitations, and the two most important are the following: (i) amphetamine hyperactivity has been interpreted as a model for a number of distinct disorders in addition to BD (including schizophrenia and drug abuse) and (ii) mania is characterized by a broad set of symptoms that may not always include motor hyperactivity (Young et al. 2011a). In two recent studies, some aspects of the cognitive deficits associated with mania have been investigated in the reverse-translated multivariate exploratory paradigm and Iowa gambling tasks in mice (van Enkhuizen et al. 2013a, b; Young et al. 2011b); however, apart from these pioneering reports, the cognitive aspects of mania were rarely studied in animal models.

A behavioral procedure that could be used to further validate a psychostimulant-induced model of mania is the ambiguous-cue interpretation test (Enkel et al. 2010), a behavioral assay that measures the cognitive judgment bias of rats in an ambiguous situation. In this procedure, the rats are trained to press a lever in an operant conditioning chamber to receive a food reward that is contingent on lever pressing in the presence of one tone and to press another lever in response to a different tone to avoid punishment by mild electric foot shock. The tones, which serve as discriminative stimuli, acquire positive and negative valence, and the training continues until the rats accomplish a stable, correct discrimination ratio. After attaining stable discrimination performance, the animals are ready to be tested. Ambiguous-cue testing comprises a discrimination task, as described above, with the presentation of additional tones that have a frequency that is intermediate between positive and negative tones. The pattern of lever press responses to this ambiguous cue is considered an indicator of the rats’ expectation of a positive or negative event (as “optimism” or “pessimism”, respectively) (for details, see Enkel et al. 2010; Papciak et al. 2013; Rygula et al. 2012, 2013, 2014).

Using this procedure, we recently demonstrated that *acute* administration of d-amphetamine induces optimism in rats (Rygula et al. 2014). As the effects of *chronic* administration of amphetamine have been widely used to model mania in laboratory animals and because mania is often associated with hyperoptimistic bias, the present study was designed to investigate how 2 weeks of chronic, daily administration of two different psychostimulants would influence the valence of the cognitive judgment bias of rats. To accomplish this goal, after initial behavioral training, two different groups of animals received daily injections of either amphetamine (2 mg/kg) or cocaine (10 mg/kg), and they were subsequently tested on the ambiguous-cue interpretation procedure. The effects of the chronic treatment were evaluated after 2 weeks of daily administration. We hypothesized that chronic amphetamine and/or cocaine treatments would increase optimism bias in rats, mimicking the behaviors observed in BD patients suffering from mania.

## Experimental procedures

### Ethics statement

These experiments were conducted in accordance with the NIH Guide for the Care and Use of Laboratory Animals and were approved by the Ethics Committee for Animal Experiments at the Institute of Pharmacology Polish Academy of Sciences.

### Subjects and housing

In total, 64 (32 in the experiment with d-amphetamine and 32 in the experiment with cocaine) male *Sprague Dawley* rats (Charles River, Germany) that weighed 175–200 g upon arrival were used in this study. The rats were group-housed (four rats per cage) in a temperature-controlled room (21 ± 1 °C) with 40–50 % humidity under a 12/12-h light/dark cycle (lights on at 0600 h). During all of the experiments, the rats were mildly food-restricted to approximately 85 % of their free-feeding weights. This goal was achieved by providing 15–20 g of food per rat per day (standard laboratory chow). The food restriction started 1 week prior to training. Water was freely available except during the test sessions. The behavioral procedures and testing were performed during the light phase of the light/dark cycle, following the protocol originally described by Enkel et al. (2010) and our previous experiments (Papciak et al. 2013; Rygula et al. 2012, 2013, 2014).

### Apparatus

The behavioral tasks were performed in eight computer-controlled operant conditioning chambers (Med Associates, St Albans, VT, USA), in which each chamber was equipped with a light, a speaker, a liquid dispenser (set to deliver 0.1 ml of 5 % sucrose solution), a grid floor through which scrambled electric shocks (0.5 mA) could be delivered, and two retractable levers. The levers were located at opposite sides of the feeder. All of the behavioral protocols, including the data acquisition and recordings, were programed in the Med State notation code (Med Associates). The experimental procedures for the ACI test used in this study were modified versions of the procedures previously described by Enkel and colleagues (2010) and have been described elsewhere (Papciak et al. 2013; Rygula et al. 2012, 2013, 2014).

### Behavioral training

#### Positive tone training

During this phase, the rats were trained to press the lever located on the left side of the feeder to receive the sucrose solution when a tone (50 s, 2,000 Hz at 75 dB) signaled the availability of a reward. Because of its association with a palatable reward, this tone acquired a positive valence and was referred to as the “positive tone,” and the associated lever was referred to as the “positive lever.” A reliable active lever pressing for the reward was achieved in three training steps: (a) Presentation of the positive tone (lasting 50 s) co-occurred with a constant (every 5 s for 5 s) delivery of the sucrose solution and was followed by a 10-s intertrial interval (ITI), (b) presentation of the positive tone co-occurred with a left lever extension and was followed by a 10-s ITI (each lever press during the tone was rewarded by sucrose solution delivery), and (c) this step was similar to (b) with the exception that after the first lever press and reward delivery, the tone was terminated and followed by a 10-s ITI. During the ITIs, the levers retracted. Each training session lasted for 30 min, and the training sessions continued until the animals attained a stable performance on each of the training steps: more than 200 responses maintained over three consecutive training sessions during step (b) and a minimum of 90 % successful responses to the positive lever following a positive tone presentation maintained over three consecutive sessions during step (c). Positive tone training was followed by negative tone training.

#### Negative tone training

During this stage, the rats were trained to press the lever located on the right side of the feeder to avoid an electric shock (0.5 mA, 10 s) when another tone (50 s, 9,000 Hz at 75 dB) signaled a forthcoming punishment. Because of its association with a concomitant punishment, this tone acquired a negative valence and was referred to as the “negative tone.” The associated lever was referred to as the “negative lever.” A reliable active lever press avoidance response was achieved in two training steps: (a) The presentation of the negative tone was accompanied by electric shocks unless the rat pressed the right (negative) lever, which terminated the shock and tone presentation and (b) the presentation of the negative tone preceded the occurrence of the electric shocks. The delay from the tone onset to the electric shock was progressively increased from 1 to 40 s. Pressing the negative lever before the shock onset terminated the tone and began a 10-s ITI, which was designated the “avoidance response.” Pressing the negative lever after the shock onset terminated the tone and shock and was referred to as the “escape response.” The maximum duration of the tone/shock application was 50 s (i.e., 40 s of tone presentation followed by 10 s of a tone/shock co-occurrence), and the tone presentations were separated by 10-s ITIs. During the ITIs, the levers retracted. Daily training sessions contained 40 tone presentations and lasted 30 min. The animals had to accomplish at least 60 % correct avoidance responses maintained over three consecutive training sessions before they were allowed to proceed to the discrimination training.

#### Discrimination training

During this phase, the rats were trained to discriminate between positive and negative tones by responding to the appropriate levers (as learned in previous training stages) to maximize reward and minimize punishment delivery. The tones, which were composed of 20 positive and 20 negative tones, were presented in non-systematic order and separated by 10 ITIs, during which the levers retracted. Pressing the positive lever during the positive tone presentation resulted in an instant reward delivery and initiated the ITI. Pressing the negative lever during the negative tone presentation resulted in a negative tone termination and initiated the ITI. Pressing the wrong lever (e.g., pressing the left lever instead of the right lever in response to a negative tone presentation), as well as escape responses or response omissions, were considered failed trials. Each training session lasted 40 min, and animals had to minimally achieve 70 % correct responses with each lever to proceed to the ACI test.

### Ambiguous-cue testing

The ACI testing session consisted of 20 positive, 20 negative, and 10 intermediate (ambiguous) tone presentations. The frequency of the intermediate tones was set to 5,000 Hz at 75 dB. This frequency was selected based on the protocol described by Enkel et al. (2010) and was confirmed to be intermediate in terms of the response pattern in a pilot experiment (data not shown). Each test lasted 50 min. The tones were presented in a non-systematic order and were separated by 10 ITIs, during which the levers retracted. Any lever press during the ambiguous tone presentation terminated the tone but had no consequences. If the rat did not respond within 50 s of the ambiguous tone presentation, the tone was terminated and a response omission was scored.

To evaluate the effects of different pharmacological treatments on the cognitive bias of animals, we measured the drug-induced changes in the optimism index. This simple measure of cognitive bias has been described elsewhere (Papciak et al. 2013; Rygula et al. 2012, 2013, 2014). To calculate the optimism index, the proportion of negative responses to the ambiguous cues was subtracted from the proportion of positive responses, which resulted in values ranging between −1 and 1, in which values above 0 indicated an overall positive judgment and “optimistic” interpretation of the ambiguous cue.

To evaluate whether the changes in the optimism index resulted from increased/decreased “optimism”/“pessimism,” the responses to each tone (positive, ambiguous, and negative) during ACI testing were scored and analyzed as the proportion of the overall number of responses to a given tone. Therefore, the increased/decreased “optimism” was defined as an increased/decreased proportion of the positive lever presses following presentation of the ambiguous cue, and the increased/decreased “pessimism” was defined as an increased/decreased proportion of the negative lever presses following presentation of the ambiguous cue. The proportion of omissions was analyzed separately.

### Drug treatment

Drugs were purchased from Sigma-Aldrich (Poznan, Poland). d-Amphetamine and cocaine were dissolved in physiological saline and administered intraperitoneally (i.p.) in a dose volume of 1 ml/kg for a period of 2 weeks. Control animals received corresponding volumes of physiological saline. The drugs and saline were injected 15 min before the ACI test sessions. The effects of drugs were tested in two separate experiments using 32 (d-amphetamine) and 32 (cocaine) animals.

#### Experimental design

After attaining a stable discrimination performance (more than 70 % correct responses to each tone over three consecutive days), the rats were subjected to three ACI tests performed at 2-day intervals (baseline), and on the basis of these tests, each cohort of 32 rats was divided into two experimental groups (control and drug-treated) matched by the average optimism index. Subsequently, drug-treated groups were subjected to daily injections of either amphetamine (2 mg/kg i.p.) or cocaine (10 mg/kg i.p.) for a period of 2 weeks, and control rats were injected daily with physiological saline (1 ml/kg i.p.) throughout the experiment. All of the drug doses used in this study were similar to doses previously applied to model BD mania in rodents (Antelman et al. 1998; Frey et al. 2006a, b). To investigate the effects of psychostimulant treatments on cognitive judgment bias, the animals were re-tested on the 14th day of treatment, 15 min after the last injection. This timing has been chosen to avoid any possibly confounding factors of drug withdrawal, which could act as a strong stressor and induce “pessimism.” The ACI tests were performed as previously described.

### Statistics

The data were analyzed using SPSS (version 20.0, SPSS Inc., Chicago, IL, USA). The effects of chronic administration of d-amphetamine and cocaine on the optimism index were investigated using two-way repeated measures ANOVA with the between-subject factor of treatment (two levels: control and drug) and with within-subject factor of test (two levels: baseline and test after 2 weeks of treatment). The effects of chronic d-amphetamine and cocaine administration on processing ambiguous cues and reference tones were investigated using four-way repeated measures ANOVA with between-subject factor of treatment (two levels: control and drug) and with within-subject factors of test (two levels: baseline and test after 2 weeks of treatment), lever (two levels: positive and negative), and tone (three levels: positive, ambiguous, and negative). For pair-wise comparisons, the values were adjusted by using Sidak’s correction factor for multiple comparisons (Howell 1997). All of the tests of significance were performed at 0.05. For repeated-measures analyses, the sphericity was verified by using Mauchly’s test. The data are presented as the mean ± SEM.

## Results

The animals reached the criteria of positive tone, negative tone, and discrimination trainings after 10 ± 0.4, 10 ± 0.2, and 25 ± 1.1 days, respectively.

In both experiments (with d-amphetamine and cocaine), the optimism index of untreated animals was significantly lower than 0 indicating pessimistic cognitive judgment bias (*t*_(31)_ = 2.864, *p* < 0.05 and *t*_(31)_ = 3.911, *p* < 0.05, respectively).

### Effects of chronic administration of d-amphetamine on the interpretation of the ambiguous cue by rats

**Fig. 1:**
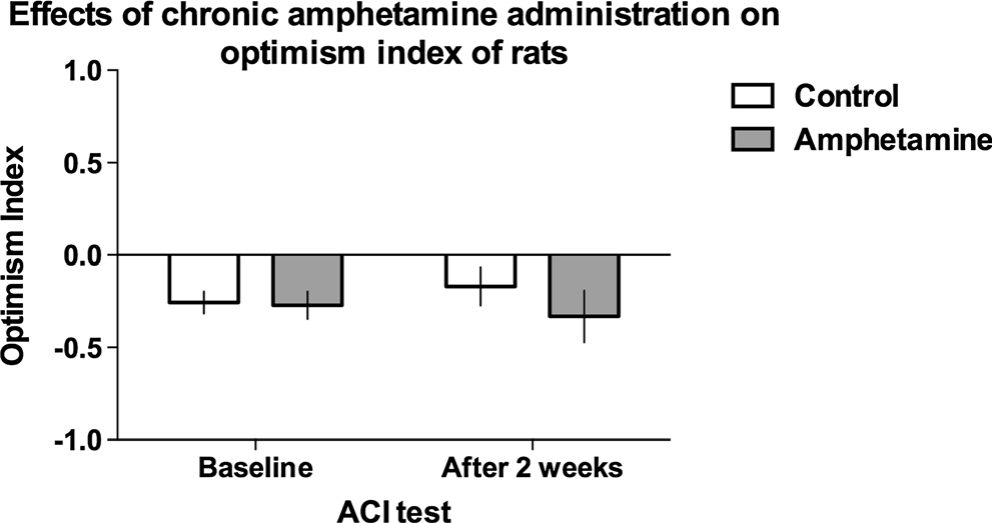
Chronic treatment with d-amphetamine (2 mg/kg/day for 2 weeks) does not change the optimism index of rats in the ambiguous-cue interpretation test. The mean ± SEM optimism index of the control (*open bars*) and amphetamine-treated (*filled bars*) rats before and after the treatment. *N* = 16 per group

As shown in Fig. 1, chronic d-amphetamine treatment (2 mg/kg/day for 2 weeks) did not have significant effects on the optimism index of rats (non-significant test × treatment interaction).

Further analysis of the lever responses after the presentation of ambiguous and reference tones revealed significant differences in the pattern of responding between control and amphetamine-treated groups after 2 weeks of treatment (test × lever × tone × group interaction (*F*_(2,60)_ = 23.77, *p* < 0.001)).

**Fig. 2:**
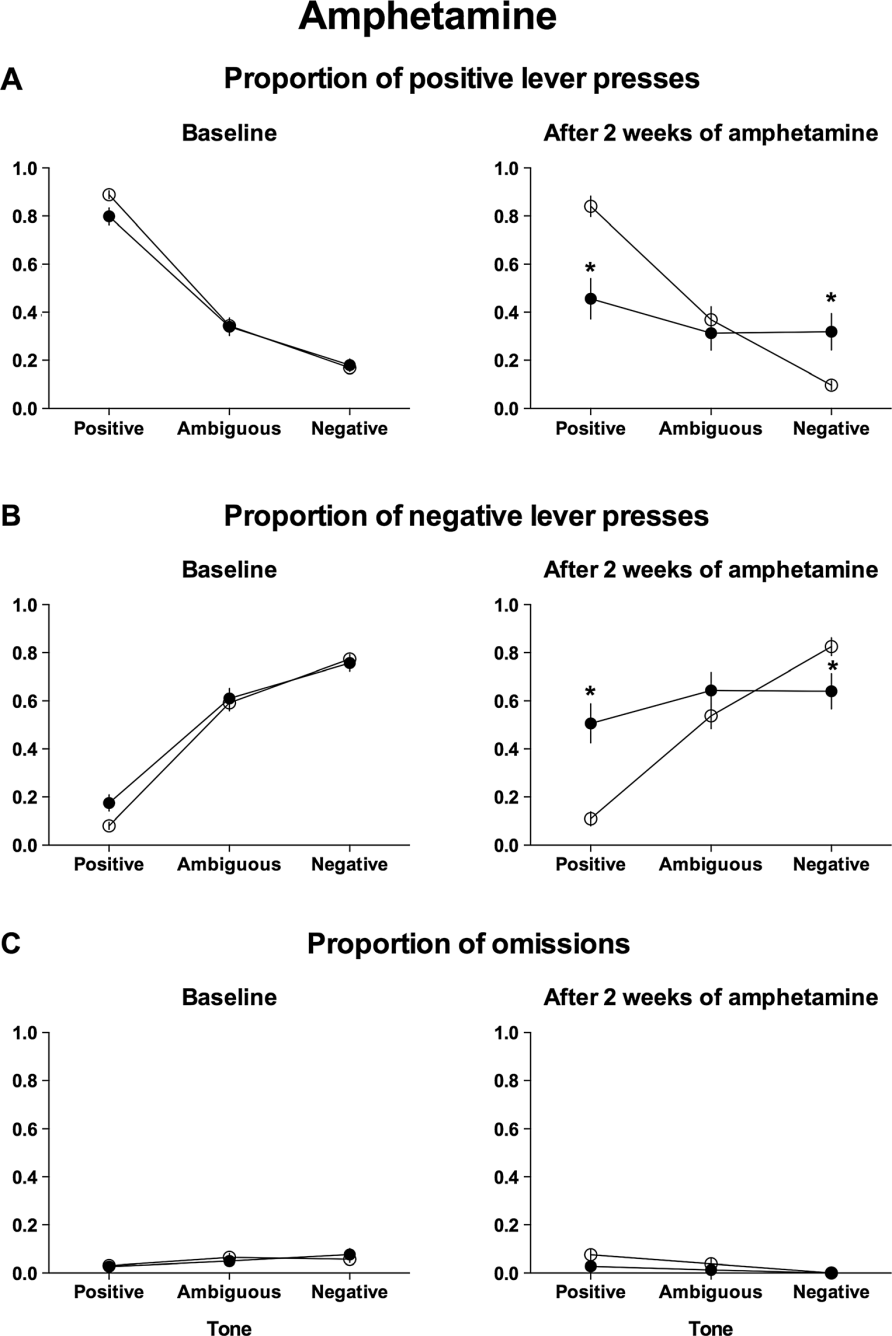
Chronic treatment with d-amphetamine (2 mg/kg/day for 2 weeks) makes rats unable to discriminate between positive and negative reference tones in the ACI test. The mean ± SEM proportion of **a** positive, **b** negative, and **c** omitted responses to the trained and ambiguous tones in the control (*open circles*) and amphetamine-treated (*filled circles*) rats. The *asterisk* indicates significant (*p* < 0.05) differences between the control and amphetamine-treated animals. *N* = 16 per group

As shown in Figs. 2a and b, after 2 weeks of amphetamine treatment, the animals responded significantly (*p* < 0.001) less often to the positive lever and significantly (*p* < 0.001) more often to the negative lever in response to the positive reference tone compared with controls and significantly (*p* < 0.001) less often to the positive lever and significantly (*p* < 0.001) more often to the negative lever in response to the positive reference tone compared with the baseline. They also responded significantly (*p* = 0.008) more often to the positive lever and significantly (*p* = 0.033) less often to the negative lever in response to the negative tone compared with their saline-treated counterparts and significantly (*p* = 0.01) more often to the positive lever and significantly (*p* = 0.022) less often to the negative lever in response to the negative tone compared with the baseline.

Analysis of the proportion of the trials in which the animals did not respond to the ambiguous and unambiguous tones revealed no significant differences between experimental groups (Fig. 2c, not significant, test × tone × group interaction).

### Effects of chronic administration of cocaine on the interpretation of the ambiguous cue by rats

**Fig. 3:**
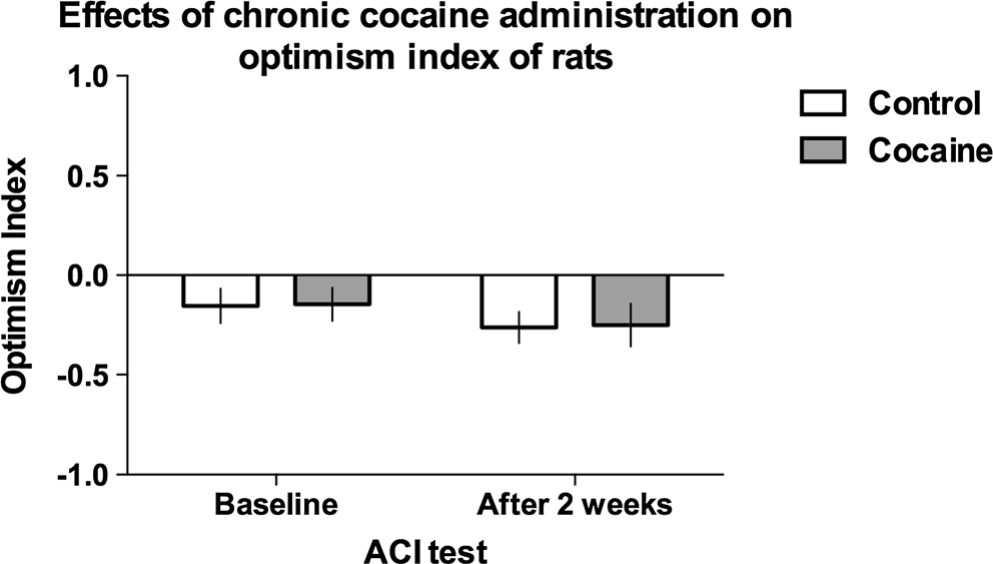
Chronic treatment with cocaine (10 mg/kg/day for 2 weeks) does not change the optimism index of rats in the ambiguous-cue interpretation test. The mean ± SEM optimism index of the control (*open bars*) and cocaine-treated (*filled bars*) rats before and after the treatment. *N* = 16 per group

As shown in Fig. 3, chronic treatment with cocaine (10 mg/kg/day for 2 weeks) also did not have significant effects on the optimism index of rats (not significant test × treatment interaction).

Analysis of the lever responding after the presentation of ambiguous and reference tones revealed significant differences in the pattern of responding between control and cocaine-treated groups after 2 weeks of treatment (test × lever × tone × group interaction (*F*_(2,60)_ = 11.65, *p* < 0.001)).

**Fig. 4:**
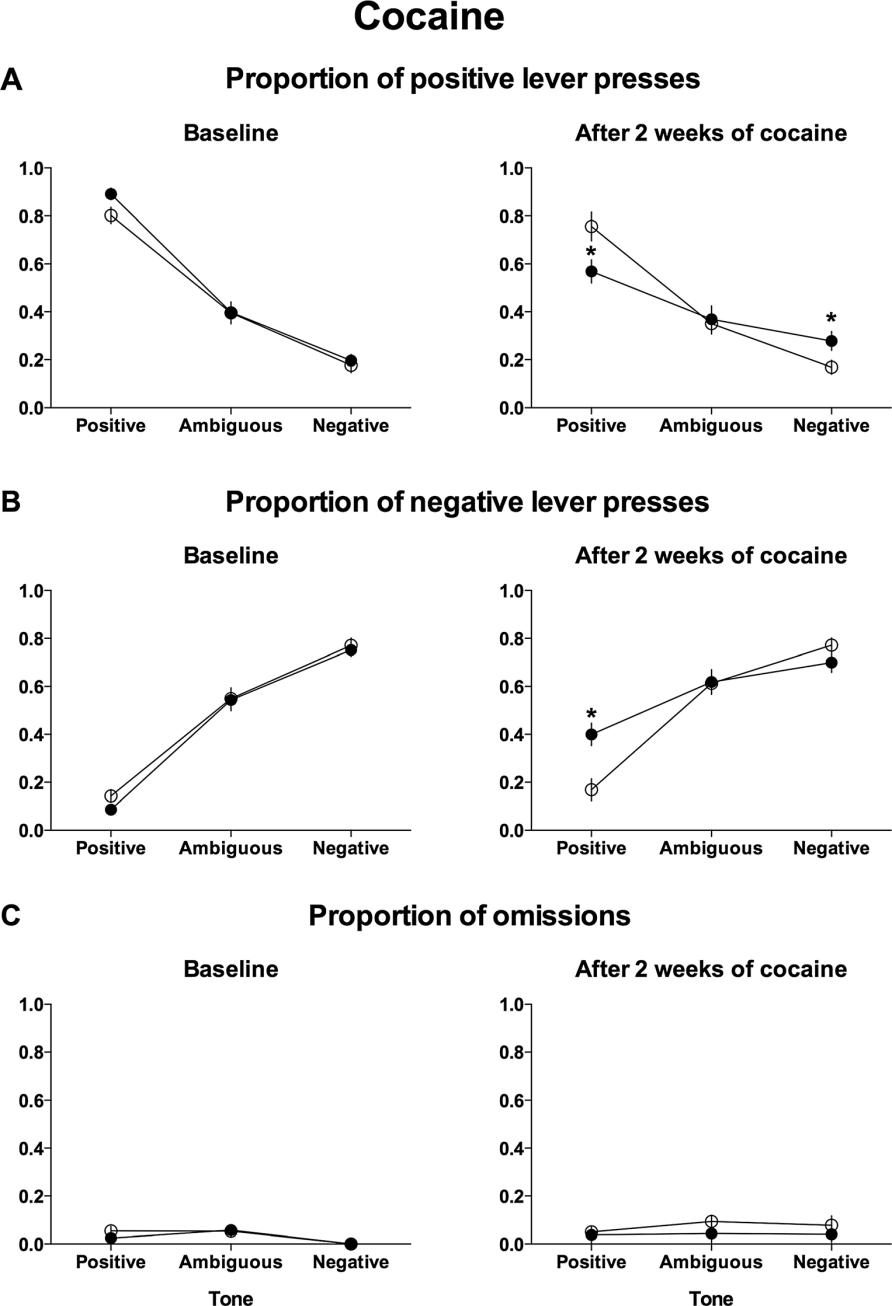
Chronic treatment with cocaine (10 mg/kg/day for 2 weeks) decreases the ability of rats to discriminate between positive and negative reference tones in the ACI test. The mean ± SEM proportion of **a** positive, **b** negative, and **c** omitted responses to the trained and ambiguous tones in the control (*open circles*) and cocaine-treated (*filled circles*) rats. The *asterisk* indicates significant (*p* < 0.05) differences between the control and cocaine-treated animals. *N* = 16 per group

As shown in Figs. 4a and b, after 2 weeks of cocaine treatment, the animals responded significantly (*p* < 0.021) less often to the positive lever and significantly (*p* < 0.001) more often to the negative lever in response to the positive reference tone compared with controls and significantly (*p* < 0.001) less often to the positive lever and significantly (*p* < 0.001) more often to the negative lever in response to the positive reference tone compared with the baseline. They also responded significantly (*p* = 0.034) more often to the positive lever in response to the negative tone compared with controls and significantly (*p* = 0.023) more often to the positive lever in response to the negative tone compared with the baseline.

Analysis of the proportion of the trials in which the animals did not respond to the ambiguous and unambiguous tones revealed no significant differences between experimental groups (Fig. 4c, not significant, test × tone × group interaction).

## Discussion

The present study was designed to investigate the cognitive judgment bias of rats in the psychostimulant-induced model of mania. Our study demonstrates that chronic administration of two potent psychostimulants, d-amphetamine and cocaine (a widely used model of mania in rodents), does not produce over-optimistic, manic-like judgment bias in rats, as measured in the ambiguous-cue interpretation test.

Instead, 2 weeks of the treatment with both drugs made animals almost completely unable to discriminate between and/or react to the positive and negative reference tones. Treated groups had a significantly decreased proportion of positive lever responses and a significantly increased proportion of negative lever responses to the unambiguous positive tone. In response to the unambiguous negative tone, the treated groups had a decreased proportion of negative lever responses and an increased proportion of positive lever responses. As the proportion of omissions made by both groups of treated animals remained unchanged, these effects of chronic psychostimulant treatments do not indicate motivational deficits. Instead, they might suggest that both treatments affected cognitive processes linked with attention, memory, and/or decision making.

Although recent studies revealed that pharmacological enhancement of DA function by acute administration of dopamine-mimetic drugs, such as l-DOPA and amphetamine, increases the optimism bias in humans (Sharot et al. 2012) and animals (Rygula et al. 2014), respectively, other studies that investigated chronic effects of psychostimulants suggested that amphetamine and cocaine can produce cognitive deficits similar to those observed after prefrontal cortex (PFC) or striatal damage. In fact, reduced postmortem levels of dopamine in the striatum and serotonin in the orbitofrontal cortex were observed in human methamphetamine and cocaine abusers (Wilson et al. 1996a, b), and chronic administration of methamphetamine has been shown to induce enduring changes in monoamine levels in the striatum and PFC of non-human primates and rats and even neurotoxic effects, including permanent axonal or nerve terminal damage (Hotchkiss and Gibb 1980; Melega et al. 1996; Ricaurte and McCann 1992; Ricaurte et al. 2005; Seiden et al. 1976, 1993; Villemagne et al. 1998). Chronic administration of d-amphetamine, in a treatment regime similar to the one used in the present study, revealed that its neurotoxic effects were associated with oxidative stress (Frey et al. 2006c, d).

Studies using behavioral procedures, such as latent inhibition, suggested that amphetamine administration increases attentional resources to irrelevant stimuli and conscious processing of irrelevant stimuli in short-term memory (Gray et al. 1992; Solomon et al. 1981; Weiner et al. 1984, 1997). d-Amphetamine has also been shown, even after single administration, to disrupt the sensory gating of auditory information (Adler et al. 1986; Stevens et al. 1991), which has been interpreted as disruption of the preattentive filtering of irrelevant sensory information. A similar effect was observed after subchronic cocaine administration (Boutros et al. 1994, 1997; Salamy et al. 1997). Given that Iowa gambling task deficits of patients with schizophrenia are associated with non-specific learning impairment (Adida et al. 2011) and that chronic psychostimulant administration has been suggested to model cognitive disruption in this disorder (Gambill and Kornetsky 1976; Tenn et al. 2003), our results could also model some aspects of schizophrenia.

Based on these reasons, the lack of effects of chronic psychostimulant treatment on the ambiguous-cue interpretation might have resulted from more general cognitive deficit and therefore must be interpreted with care. Without proper discrimination and reaction to the reference positive and negative tones, measurements of the cognitive judgment bias based on the ambiguous-cue interpretation may not be reliable.

As observed during the baseline tests, the animals used in the present study were generally pessimistic (negative basal optimism index). Because we have shown recently that the valence of the cognitive judgment bias in rats, similar to humans, has both enduring trait and transient state components (Rygula et al. 2013) and that rats displaying the “pessimistic” trait were more prone to develop stress-induced anhedonia compared to their “optimistic” conspecifics, we cannot exclude the possibility that this initial pessimism also interacted with the effects of psychostimulants and perhaps abolished them. Clearly, further studies investigating how individual differences in the interpretation of the ambiguous cue interact with pharmacological treatments are required to test this hypothesis.

As discussed elsewhere (Rygula et al. 2012), contrary to previous studies (Enkel et al. 2010; Harding et al. 2004), we did not investigate different degrees of ambiguity. The experimental design with only one ambiguous tone allowed us to apply a reasonably high (10) number of these stimuli in only one testing session, and the 50 discriminations per session provided a broad and precise scale for the valence of responding.

Finally, as we tested the animals only 15 min after the last drug injection, it is likely that their behavior was affected by some non-specific drug actions such as hyperactivity or stereotypies. Indeed, previous studies, using a treatment regime similar to the one applied in the present study, indicated psychostimulant-induced sensitization in the locomotor activity (Frey et al. 2006a, c). This, however, adds to the validity of the test, as psychostimulant-induced hyperactivity is considered one of the main behavioral correlates of mania in rodents (Young et al. 2011a).

## Conclusion

Whereas animal models of other psychiatric disorders, such as depression, have proved already to be a vital tool in preclinical research, the development of new models of mania remains a challenging task. As with all animal models of psychiatric conditions, inferring affective states in rodents that mirror the variety of clinical symptoms and cognitive deficits that characterize this human psychopathology is difficult.

In the present study, we assumed the challenge of modeling cognitive aspects of mania in animals. Although our study clearly shows that chronic treatment with two psychostimulant drugs (amphetamine and cocaine) do not produce manic-like, over-optimistic cognitive judgment bias in rats, the results might have been obscured by a variety of non-specific effects of chronic psychostimulant treatment. Further studies using a combination of the ACI test with genetic models of dopaminergic hyperactivity will help to avoid those non-specific effects and may allow investigation of the cognitive judgment bias associated with mania in animals.
